# Single-cell transcriptomics in ovarian cancer identify a metastasis-associated cell cluster overexpressed RAB13

**DOI:** 10.1186/s12967-023-04094-7

**Published:** 2023-04-12

**Authors:** Jiahao Guo, Xiaoyang Han, Jie Li, Zhefeng Li, Junjie Yi, Yan Gao, Xiaoting Zhao, Wentao Yue

**Affiliations:** Central Laboratory, Beijing Maternal and Child Health Care Hospital, Beijing Obstetrics and Gynecology Hospital, Capital Medical University, Beijing, 100026 China

**Keywords:** Ovarian cancer, Metastasis, Single-cell transcriptomics, RAB13, Heterogeneity

## Abstract

**Background:**

Metastasis, the leading cause of cancer-related death in patients diagnosed with ovarian cancer (OC), is a complex process that involves multiple biological effects. With the continuous development of sequencing technology, single-cell sequence has emerged as a promising strategy to understand the pathogenesis of ovarian cancer.

**Methods:**

Through integrating 10 × single-cell data from 12 samples, we developed a single-cell map of primary and metastatic OC. By copy-number variations analysis, pseudotime analysis, enrichment analysis, and cell–cell communication analysis, we explored the heterogeneity among OC cells. We performed differential expression analysis and high dimensional weighted gene co-expression network analysis to identify the hub genes of C4. The effects of RAB13 on OC cell lines were validated in vitro.

**Results:**

We discovered a cell subcluster, referred to as C4, that is closely associated with metastasis and poor prognosis in OC. This subcluster correlated with an epithelial–mesenchymal transition (EMT) and angiogenesis signature and RAB13 was identified as the key marker of it. Downregulation of RAB13 resulted in a reduction of OC cells migration and invasion. Additionally, we predicted several potential drugs that might inhibit RAB13.

**Conclusions:**

Our study has identified a cell subcluster that is closely linked to metastasis in OC, and we have also identified RAB13 as its hub gene that has great potential to become a new therapeutic target for OC.

**Supplementary Information:**

The online version contains supplementary material available at 10.1186/s12967-023-04094-7.

## Introduction

Ovarian cancer (OC) has the worst prognosis and highest mortality rate among gynecological cancers, causing about 200,000 deaths annually worldwide [1]. Despite the recent advances in the treatments of OC, the 5-year survival rate of OC remains below 50% owning to its high recurrence and metastasis rate [2]. Most of the deaths result from recurrence with metastasis [3]. Therefore, it is imperative to develop new treatments against cancer metastasis.

The tumor heterogeneity is prevalent among cells and contributes to tumor differentiation into different cell subtypes, interacting with the tumor microenvironment (TME), and finally leading to metastasis [4]. Intratumoral heterogeneity (ITH) closely correlated to the invasive and metastatic ability of tumors, and impacts the clinical diagnosis and treatment of patients. A series of studies have revealed the heterogeneity and explored the mechanism of OC at bulk sequence level [5, 6]. But the traditional bulk sequence methods just focus on the tissue resolution in studying ITH. Single-cell sequence technology detects heterogeneity of tumor cells by single-cell resolution, identifies rare cells, delineates cell subclusters, tracks cell lineages, locates mutated genes, and discovers new biomarkers, providing us with a new perspective to study tumor metastases [7]. Benjamin et al. revealed a single cell landscape of high grade serous OC and developed molecular subtypes among patients, which helps guide individualized treatment [8]. Tang et al. conducted a multiomic study on ovarian cancer and found that tumor heterogeneity was strong in OC [9]. In addition, a series of studies have explored the mechanisms and treatment of OC at single cell resolution [10–12]. However, limited by the sample size and the high ITH of OC, few of them focused on the commonalities among multiple patients.

In this study, through integrating of single-cell sequence data from 12 patients, we constructed a cell atlas containing normal epithelium, primary carcinoma, and metastatic carcinoma. The cancer cell developmental trajectory of metastasis was described by pseudotime trajectory analysis at different stages, and a cell subcluster with commonality in patients and closely associated with metastasis was discovered. Cell–cell communication analysis revealed the immune escape and pro-mesenchymal growth properties of this subcluster. Based on it, an overexpressed gene named RAB13 that had not been reported in OC was identified. We further verified the effects that RAB13 promoting metastasis on OC cell lines, which showed that RAB13 promoted cell migration and invasion in vitro. Then, we explored the relationships between the expression level of RAB13 and clinical phenotypes with TCGA datasets, which indicated RAB13 was significantly associated with worse prognosis and tumor progression. Finally, two cytoskeleton inhibitors that may target RAB13 were predicted. In conclusion, our study helps to further explore the mechanisms for OC metastasis, and provides a new potential treatment target of OC.

## Materials and methods

### Collection of OC and normal ovary samples

Tissues from one patient with ovarian cancer and one with normal ovary sample were collected for single cell suspension preparation. The fresh tissues were collected at the time of resection and then transported by MACS Tissue Storage Solution (MACS, Cat. no.130-100-008F) on ice. Tissues were subsequently washed for 2–3 times with phosphate buffered saline (PBS; Hyclone, Cat. no. SH30256.01) and minced on ice. The Tumor Dissociation Kit (MACS, Cat. no.130-095-929) was used to digest the tissues in order to prepare single-cell suspensions. Next, the tissues were dissociated at 37 °C with a shaking speed of 30 rpm for 6 min. The dissociated cells were digested and collected with 0.25% trypsin (Gibco, Cat. No. 25200056) for 2 min. Cell suspensions were filtered using a 40 μm nylon cell strainer (Falcon, Cat. no. 352340), the red blood cells were thus removed. Single-cell suspensions were stained with AO/PI fluorescent dyes (Logos Biosystems, Cat. no. LB F23001) to check viability with LUNA (Logos Biosystems, Cat. no. LUNA-STEM). And then diluted them to approximately 1 × 106 cells/ml with PBS containing 0.02% BSA for single-cell sequencing. Cells were loaded according to the standard protocol of the Chromium single cell 3′ kit, capturing 5,000 cells to 10,000 cells/chip position (V3 chemistry). Libraries for scRNA-seq were generated using the 10 × Genomics Chromium platform and sequenced on an Illumina Novaseq 6000 system.

### Public data sources

The public scRNA-seq datasets were downloaded from Gene Expression Omnibus database [13], with accession numbers GSE154600, GSE158937 and GSE181955 (Additional file 5: Table S1). Moreover, the bulk RNA-seq expression dataset and phenotype dataset of TCGA ovarian cancer were downloaded from UCSC Xena [14, 15].

### Quality control and data integration

Further quality control was applied to cells: cells were filtered for detected genes (min: 300-max: 6000), mitochondrial gene percent (0–15%), hemoglobin gene percent (0–0.1%) and ribosomal gene percent (min: 1–100%). Then genes expressed in less than 3 cells were removed as well. The two samples with low data quality (due to their high mitochondrial gene percent and unusual cell proportion), GSM4675274 and GSM4816046, were removed. Subsequently, cells were integrated by CCA method, using ‘IntegrateData()’ function of R package ‘Seurat’ [16].

### The chromosomal copy-number variations estimation

The chromosomal copy-number variations (CNVs) were estimated by R package ‘inferCNV’ [17]. B cells, T cells, myeloid cells, endothelial cells, fibroblasts and benign epithelial cells were used as reference. The CNVs scores were obtained by accumulating the CNVs levels of cells within each subcluster.

### Gene set functional analysis

The gene set functional analyses were conducted with R package ‘clusterProfiler’ [18] and ‘GSVA’ [19]. Gene Ontology (GO), Kyoto Encyclopedia of Genes and Genomes (KEGG) and Reactome pathway database were used for GSVA analyses. Hallmark gene sets and Reactome gene sets were obtained from R package ‘msigdbr’ [20].

### Survival analysis

The top 10 marker genes for each cell subcluster were extracted. Subsequently, subcluster feature scores of the 379 OC patients from the TCGA cohort were calculated by GSVA. Combining with the overall survival time, we finally performed a Kaplan–Meier survival analysis by R package ‘survival’.

### Pseudotime analysis

Single-cell pseudotime trajectory was constructed using R package ‘monocle3’ (monocle3 v1.0.1) [21]. UMAP method was applied to reduce dimensions, and function of ‘plot_cells’ was used for visualization. The ‘graph_test’ function was used to screen for DEGs. The threshold of Morans index was set at > 0.3 and the q-value (corrected p value) threshold was set at < 0.001.

### Cell–cell communication analysis

We used R package ‘CellChat’ (CellChat v1.1.3) to perform cell–cell communication analysis [22]. For analysis, 500 cells from each cell subcluster were selected randomly by function ‘subset’. Cellchat database including ‘Secreted Signaling’, ‘ECM-Receptor’ and ‘Cell–Cell Contact’ were used. The minimum cell count was set at 10 for filtering.

### HdWGCNA analysis

High dimensional weighted gene co-expression network analysis (hdWGCNA) was used to construct a scale-free network at single cell level by R package ‘hdWGCNA’. After set the threshold of scale-free topology model fit as > 0.85, soft threshold was selected as 5 for the best connectivity. GSVA was used to score TCGA cohort with modules. Correlations between modules and phenotype were evaluated by Spearman test. The function ‘HubGeneNetworkPlot’ was used to construct a protein–protein interaction (PPI) network.

### Differential expression analysis

Five hundred cells from primary and metastatic samples were selected by random sampling. Then differential expression analysis was performed by R package ‘DESeq2’. Genes with log_2_FoldChange absolute values > 1 and adjusted p value < 0.01 were regarded as differential expressed genes (DEGs).

### Drug sensitivity prediction

We performed the prediction analysis of drug sensitivity of the commonly used or potential chemotherapy drugs for OC by R package “pRRophetic”. TCGA cohort including 379 patients was divided into two groups by the median of gene module GSVA score.

### Quantitative real-time PCR (RT-qPCR)

Total RNA was isolated using the kit (Invitrogen, Calsbad, CA). Next, cDNAs were synthesized according to protocols (Toyobo, Shanghai, China). Then, qRT-PCR was conducted on an ABI 7500 Real-Time PCR system (Applied Biosystems, Foster City, USA). qRT-PCR primers for RAB13 were as follows: forward primer 5′-ACATCTCCACCATCGGAATTGAT-3′ and reverse primer 5′-TGTCTTGAACCGCTCTTGGC-3′. Finally, the relative expression of the genes was analyzed by the comparative 2^−ΔΔCT^ method.

### Cell culture and small interfering RNA (siRNA) transfection

Human OC cell lines SK-OV-3, A2780 and ovarian epithelial cell line OSE were obtained from ATCC. SK-OV-3, A2780 and OSE cell lines were maintained in RPMI-1640 (Gibco, MD, USA), supplemented with 10% FBS (HyClone, USA) and 100 μg/ml streptomycin/penicillin (Gibco, MD, USA) under standard culture conditions (5% CO2, 37 °C). After the cell lines reached a confluency of 50–60%, they were transfected with 50 pmol/mL of siRNAs using lipofectamine 3000 (Invitrogen, Carlsbad, CA, USA) for 8 h. Then, the transfection system was removed and cells were cultured under standard culture conditions. Protein analysis and functional experiments were performed digested cells 48 h after seeding. RNA analysis was performed on harvested cells 24 h after seeding. The siRNAs against RAB13 were obtained from JTSBIO Co., Ltd. (Wuhan, China) and the sequences of siRAB13 were as follows: si1 (sense: 5′-CAAGAGGAAGGUGCAGAAGTT-3′; anti-sense: 5′-CUUCUGCACCUUCCUCUUGTT-3′); si2 (sense: 5′-GUGACAAGAAGAACACCAATT-3′; anti-sense: 5′-UUGGUGUUCUUGUCACTT-3′).

### Western blot and antibodies

RIPA buffer (Thermo Fisher Scientific, Waltham, MA, USA) was used to extract protein from cell samples. The protein was measured using BCA assay (Thermo Fisher Scientific, Waltham, MA, USA). An equal amount of 30 µg of protein was added to each sample and subsequently separated by SDS-PAGE (sodium dodecyl sulfate–polyacrylamide gel electrophoresis). After transferred onto polyvinylidene fluoride membranes (Gene Molecular Biotech, Inc., Shanghai, China), membranes were blocked with 5% nonfat milk in TBST for 2 h at room temperature. Then were incubated with primary antibodies at 4 °C overnight, as following dilutions: anti-RAB13 (1:2000, ab180936, Abcam), anti-GAPDH (1:5000, KC-5G5, KangChen). Next, the membranes were incubated with horseradish peroxidase-conjugated rabbit IgG secondary antibodies (1:5000, CST) for 1 h at room temperature. The expression levels were detected by ECL kit (Roche Diagnostics, Basel, Switzerland) using WB imaging system.

### Wound-healing assay

The transfected cells were cultured in a 6-well plate to 100% confluence and then scribed vertically using a micropipette tip. Then the cell culture was changed to serum-free culture in order to exclude proliferation effects. The wound healing rate was observed and photographed 48 h later.

### Transwell migration and invasion assays

We performed Transwell assays using 24-well plates with Transwell chamber system (Corning, USA). The upper chambers were plated with 100 μL Matrigel and placed at 37 °C for 12 h for the invasion assay. 3 × 10^4^ transfected cells were inoculated in the upper chamber with 200μL serum-free culture medium. 600μL culture medium with 20% FBS was plated in the lower chamber. After incubation at 37 °C, 5% CO^2^ for 48 h (24 h for the migration assay), the cells in upper chamber were washed off and the chamber was immersed in 4% crystal violet for 15 min. Then migrated or invaded cells were photographed under microscope. Four randomly selected views were counted for statistical analysis per well.

## Results

### Single-cell RNA sequence data integration and clustering

**Fig. 1:**
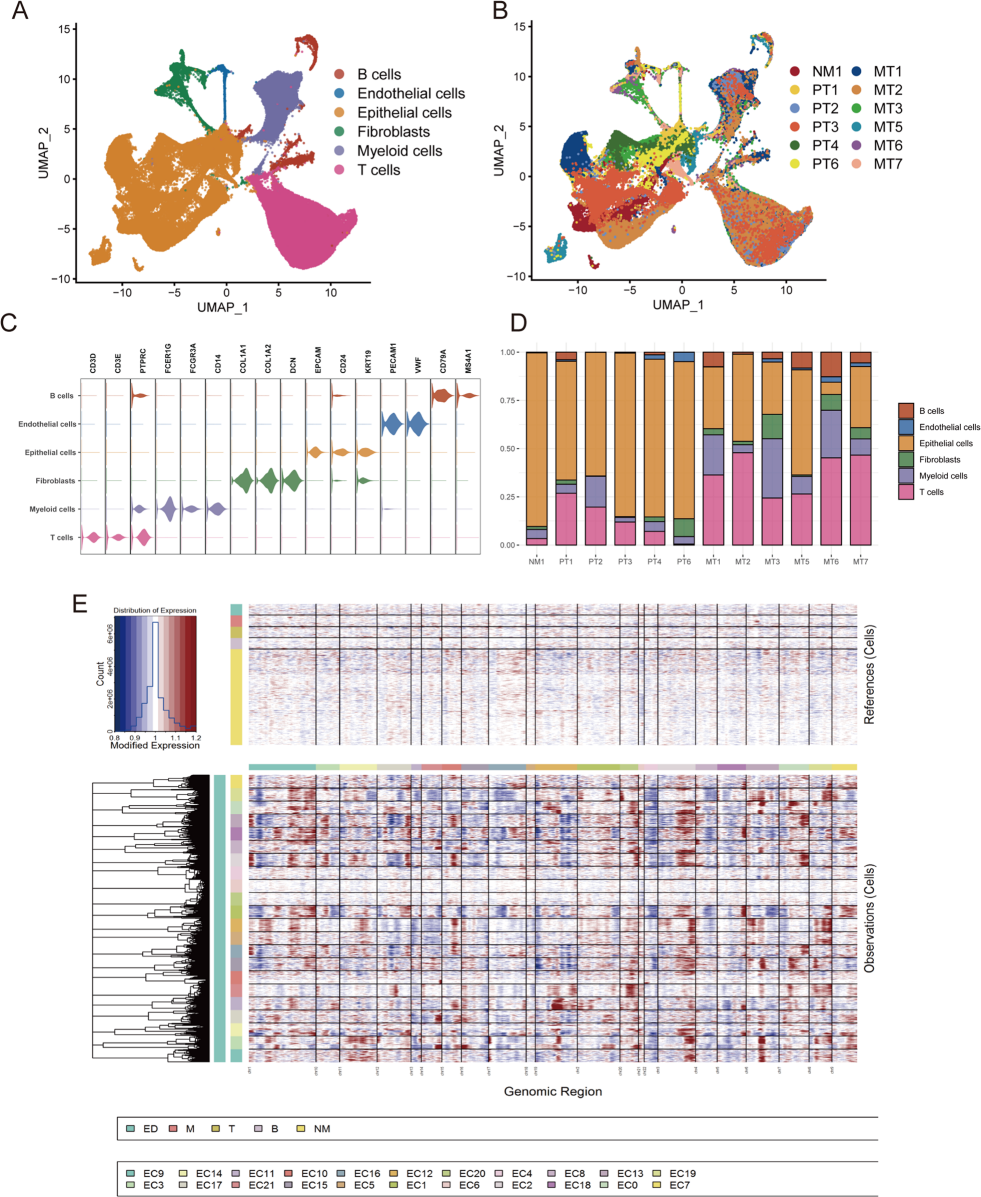
Single-cell atlas of 12 patients. **A**, **B** UMAP of the all 155,173 cells. Colored by cell type or patient. NM: normal epithelium; PT: primary tumor; MT: metastatic tumor. **C** Violin plot showed the markers of each cell type. **D** Bar plot showed the cell proportion among patients. **E** Chromosomal landscape of inferred CNVs among cancer cell subclusters

For subsequent analysis, scRNA-seq data from 12 patients (including six omental metastatic tissues, five primary ovarian cancer and one normal ovarian epithelium) were integrated by canonical correlation analysis (CCA). A total of 155,173 cells were acquired after quality control and filtering. After defining the numbers of principal components (nPCs = 30) and resolution (resolution = 0.2), uniform manifold approximation and projection (UMAP) method was used for non-linear dimension reduction and 18 cell clusters were identified (Additional file 1: Fig. S1). Based on canonical markers, six cell types from 12 patients were finally separated (Fig. 1A, B: B cells (markers: CD79A, MS4A1), endothelial cells (markers: PECAM1, VWF), epithelial cells (markers: EPCAM, CD24, KRT19), fibroblasts (markers: COL1A1, COL1A2, DCN), myeloid cells (markers: FCER1G, FCER3A, CD14) and T cells (markers: CD3D, CD3E) (Fig. 1C).

Interestingly, with the progress of the disease, the composition of epithelial cells was decreasing, while the composition of immune cells and mesenchymal cells was increasing relatively. It was in line with our conventional understanding that metastatic tumors have a more complex TME (tumor microenvironment) (Fig. 1D). This suggested that the smaller proportion of metastatic cancer cells may have a more aggressive malignant behavior compared with primary cancer cells.

### Heterogeneity between cancer cell clusters of OC

**Fig. 2:**
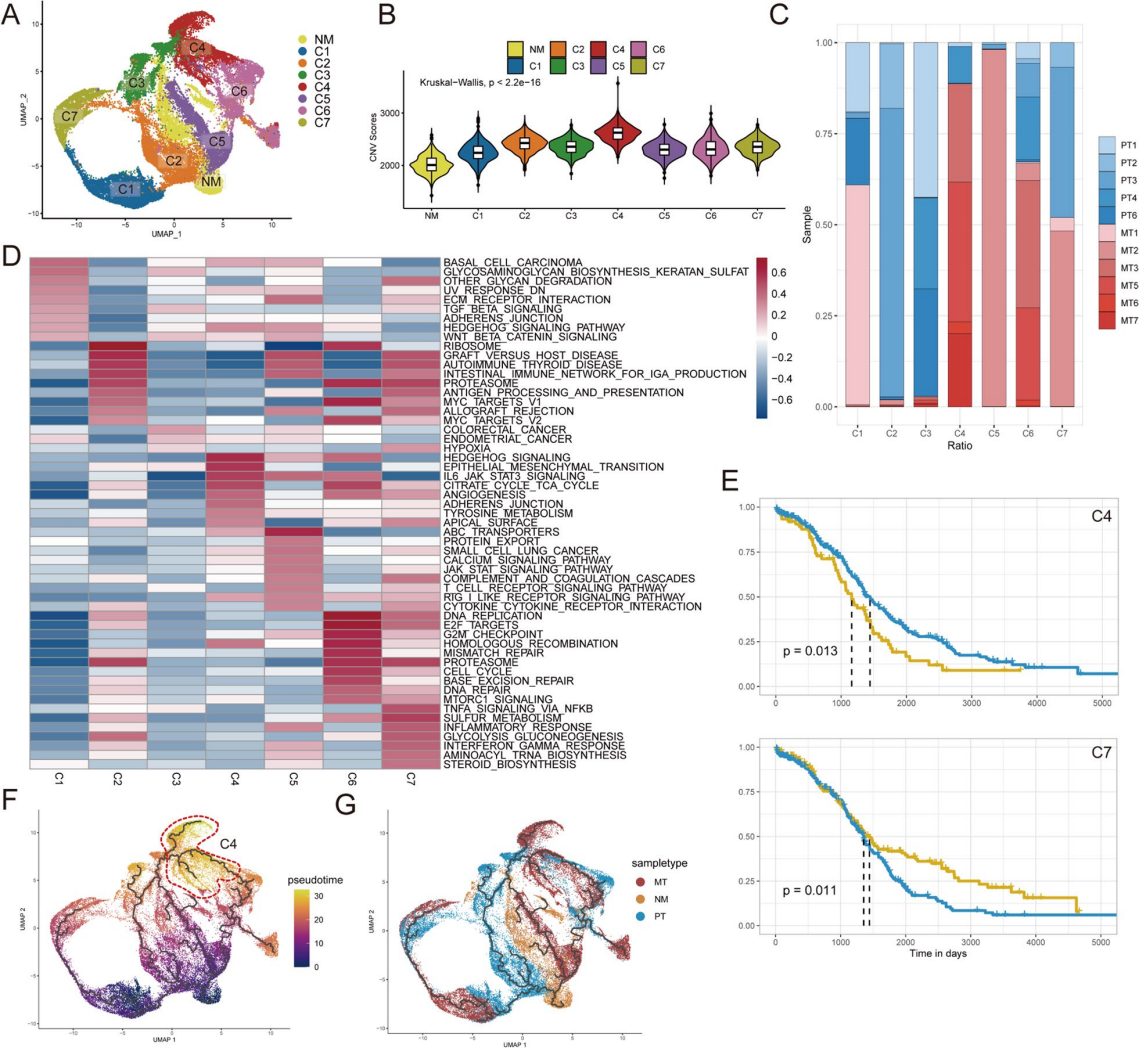
Heterogeneity of transcriptomes among OC cells. **A** Dimplot showed the UMAP of epithelial cell subclusters. **B** Violin plot demonstrated the difference in CNV scores among benign and malignant cell subclusters. **C** Malignant cell proportion among patients. **D** Hallmark and Reactome pathways of malignant cell subclusters determined by GSVA. **E** Kaplan–Meier analysis for patients from TCGA cohort with high and low GSVA score based on the top 20 markers of C4 (top) and C7 (bottom). **F**, **G** Pseudotime trajectory analysis highlights subcluster C4 that mainly derived from metastatic samples with the largest pseudotime

Next, we performed inferring chromosomal copy-number variations (CNVs) analysis among all epithelial cells with reference cells of endothelial cells, immune cells (myeloid cells, T cells and B cells) and normal epithelial cells (Fig. 1E). Based on the CNV levels, malignant cells were separated from all epithelial cells. Then, the malignant cells were reclustered into seven subclusters named C1 to C7 by UMAP analysis according to the similarity of gene expression (Fig. 2A). The CNV scores in most malignant cells were obviously higher than that in normal epithelial cells. In addition, C4 has the highest CNVs score among all subclusters (Fig. 2B and Additional file 2: Fig. S2), which suggests that C4 may have a higher malignant potential than other subclusters. As is shown in Fig. 2C, subclusters C2 and C3 were mainly presented in primary sites; C1, C6 and C7, contained cells from the primary sites and the metastatic sites; except them, C4 mainly existed in metastatic sites and C5 was a patient-specific subcluster from only one metastatic site. This indicated that the malignant cells were highly spatially heterogeneous, while we were aiming to explore the commonalities of metastatic ovarian cancer from different patient sources.

To explore the functions among the subclusters, we then performed a gene set variation analysis (GSVA). The results showed that different cancer subclusters were associated with diverse biological processes (Fig. 2D). C2, C5 and C7 enriched immunity related pathways such as ‘antigen process and presentation’, ‘inflammatory response’, ‘cytokine-cytokine receptor interactions’ and ‘interferon gamma response’. Therefore, we regarded them as immune-active subclusters. C4 existed with an invasive signature including malignant biological properties such as Hedgehog signaling, epithelial–mesenchymal transition, angiogenesis, and adherence junction, which were previously considered to be correlated with angiogenesis, chemotherapy resistance and tumor invasion [23, 24]. C6 enriched proliferative and DNA repair pathways such as ‘cell cycle’, ‘G2M checkpoint’, ‘DNA repair’ and ‘homologous recombination’, indicating it was at a high proliferative status. As described in previous study, the proliferative DNA repair subcluster C6 was an untreated cell cluster which had better chemotherapy response and prognosis [25]. Thus, functional analysis revealed that C4 was an invasion-associated subcluster. Studying this subcluster may help us understand the mechanism of OC metastasis and develop new therapeutic targets.

Then, using the bulk RNA-seq data and clinical data from The Cancer Genome Atlas (TCGA), we investigated the relationship of subclusters with patient prognosis (Additional file 3: Fig. S3). Among the 7 subclusters identified from these 12 patients, only two subclusters were significantly linked to prognosis, of which C4 was negatively associated with overall survival (OS) time (log-rank test, p = 0.013), while C7 was positively associated with OS time (log-rank test, p = 0.011) (Fig. 2E). To investigate the transition between benign epithelium, primary cancer and metastatic cancer, we performed a pseudotime trajectory analysis of all epithelial cell groups. Through placing the normal epithelial cells at the beginning of the trajectory, cells were separated by cancer progression (Fig. 2F, G). We suggested that the ends of pseudotime trajectories including C3, C4 and C7 were different end states of cancer cells. Based on this, we obtained three main transition trajectories and identified 532 differentially expressed genes of them (Additional file 6: Table S2). We also considered that pseudotime trajectory analysis was susceptible to the interindividual variation [25], but most subclusters except for C5 were derived from multiple samples (Fig. 2C). In this section, we explored the heterogeneity among OC cells and identified a specific OC cell subcluster, C4, which was related to metastasis and worse prognosis.

### C4 promotes tumor metastasis through cell–cell communication with mesenchymal cells

**Fig. 3:**
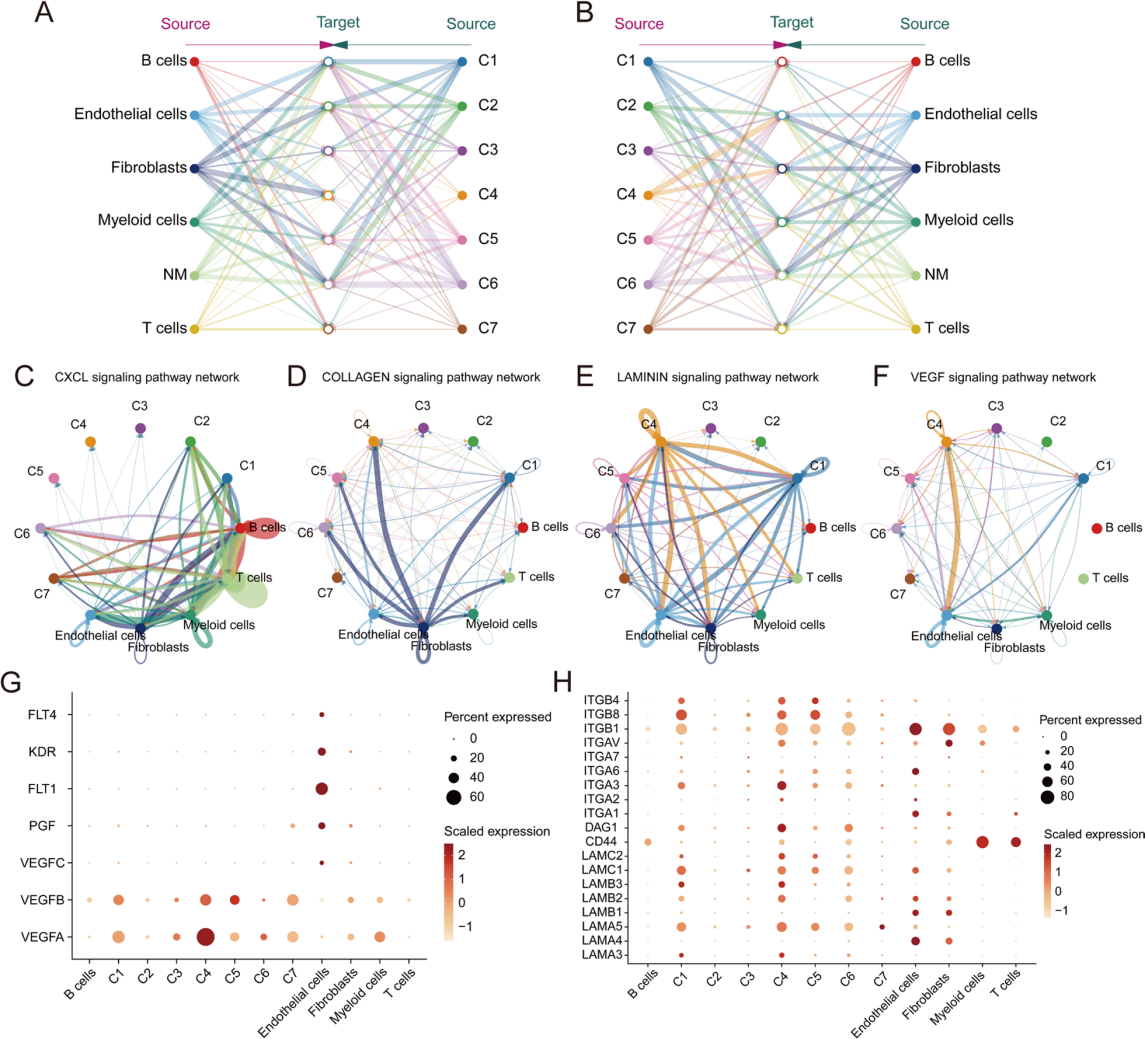
Cell–cell Communications between OC and TME cells. **A**, **B** Cell communications among cancer cell subclusters and TME. The thickness of the line indicates numbers of signaling targeting malignant cells or TME cells. **C**–**F** Circle plots demonstrating the interactions of CXCL, Collagen, Laminin and VEGF pathways. **G**, **H** Bubble plots showing the ligands and receptors of VEGF and Laminin pathways

We then performed cell–cell communication analysis to elucidate the interactions of different subclusters and the TME cells. The results showed that most of the tumor cells except for C7 had a low number of cell interactions with immune cells (including B cells, T cells, and myeloid cells). This indicated that C7 was an immune-activated subcluster, corroborating with its previously mentioned association with a better prognosis. Meanwhile, C4 had the biggest cell interaction number with mesenchymal cells (including vascular endothelial cells and fibroblasts) and few interactions with immune cells (Fig. 3A, B), suggesting that C4 may have a higher level of immune escape and promote angiogenesis and tumor invasion through cellular communication with mesenchymal cells. Furthermore, we found that pathways related to chemokines, cytoskeleton and angiogenesis, including CXCL, Laminin, Collagen and VEGF, ranked highest in contribution, besides pathways common to all subgroups such as MK and SPP1 (Additional file 4: Fig. S4). For the CXCL pathway, C1, C2, C6 and C7 all had more communications with immune cells, suggesting that they may have a better immune response (Fig. 3C). In contrast, C4, C5, and C6 did not produce signals in the immune related pathway, and their communications focused on the Laminin and Collagen pathways interacting with fibroblasts (Fig. 3D, E). In addition, the VEGF pathway that mainly interacted with endothelial cells was much stronger in C4 than that in the other six subclusters (Fig. 3F). We also identified the receptor-ligand interactions of these pathways (Fig. 3G, H). We noticed C4 expressed higher levels of VEGFA and VEGFB, while its receptors, FLT1, PGF and KDR were specifically expressed in endothelial cells, indicated that C4 was a subcluster promoting angiogenesis and metastasis. Moreover, C4 significantly overexpressed the laminin family genes, which were considered correlating to extracellular matrix (ECM) remodeling and tumor invasion [26]. In summary, our work indicated that C4 was an invasive subcluster, promoting angiogenesis, ECM remodeling and tumor metastasis through cell–cell communication.

### HdWGCNA identifies the hub genes of C4 related to metastasis

**Fig. 4:**
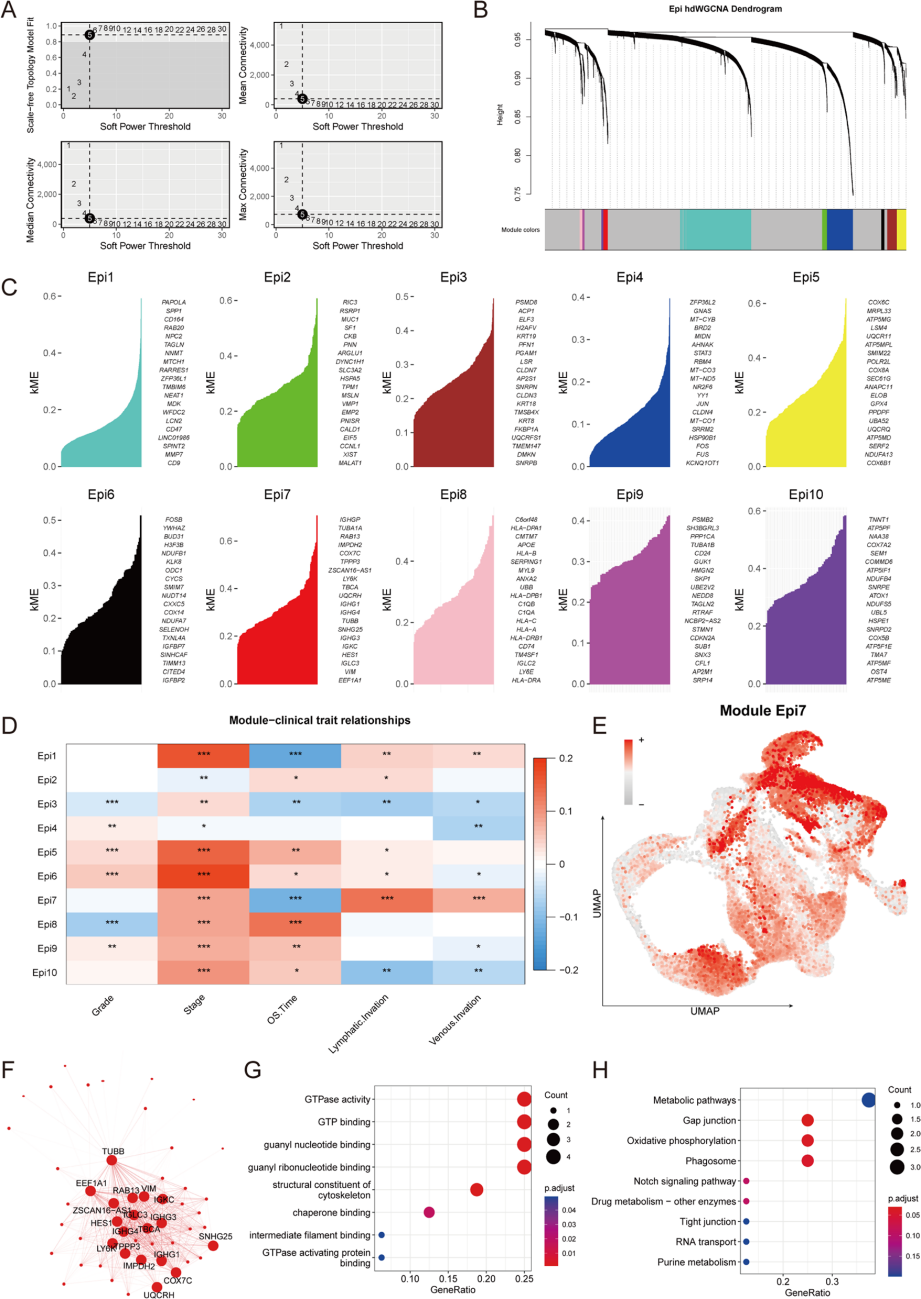
Identification of gene co-expression modules among OC cells. **A**, **B** Weighed gene co-expression network analysis was constructed among malignant cells. (See “Materials and methods” section) **C** The first 20 eigengenes of each module, ranked by eigengene-based connectivity (kME). **D** Heatmap showed the relationships between modules and clinical phenotypes. **E** UMAP of the expression of Epi7 among all epithelial cells. **F** Protein–protein interaction network demonstrated the interactions within Module Epi7. **G**, **H** Dot plot of the GO (G) and KEGG (H) functional enrich analysis of the module Epi7. (*p < 0.05, **p < 0.01, ***p < 0.001 in a spearman test.)

Next, high dimensional weighted gene co-expression network analysis (hdWGCNA) was used to identify the main molecular characteristics of C4. With a soft threshold of 5, the scale-free network of C4 was constructed for the best connectivity and a total of 10 gene modules were identified (Fig. 4A–C). The module Epi7 had the strongest correlation with lymphatic and venous invasion and was negatively correlated with overall survival time (Fig. 4D). The GSVA scores of Epi7 module among all epithelial cells were calculated, the result showed that C4 scored the highest (Fig. 4E). We then constructed a PPI network, demonstrating the hub genes and the interactions of module Epi7 (Fig. 4F), the GTPase, microtubules, microfilaments and cytoskeleton related genes such as TUBB, RAB13 and VIM were in a central position. Then we performed GO and KEGG enrichment analyses for Epi7, the functions mainly focused on cytoskeleton and GTPase related pathways (Fig. 4G, H). In conclusion, we explored the gene expression modules within C4, and identified the hub genes leading to metastasis.

### Downregulation of RAB13 suppresses migration and invasion abilities of OC cells

**Fig. 5:**
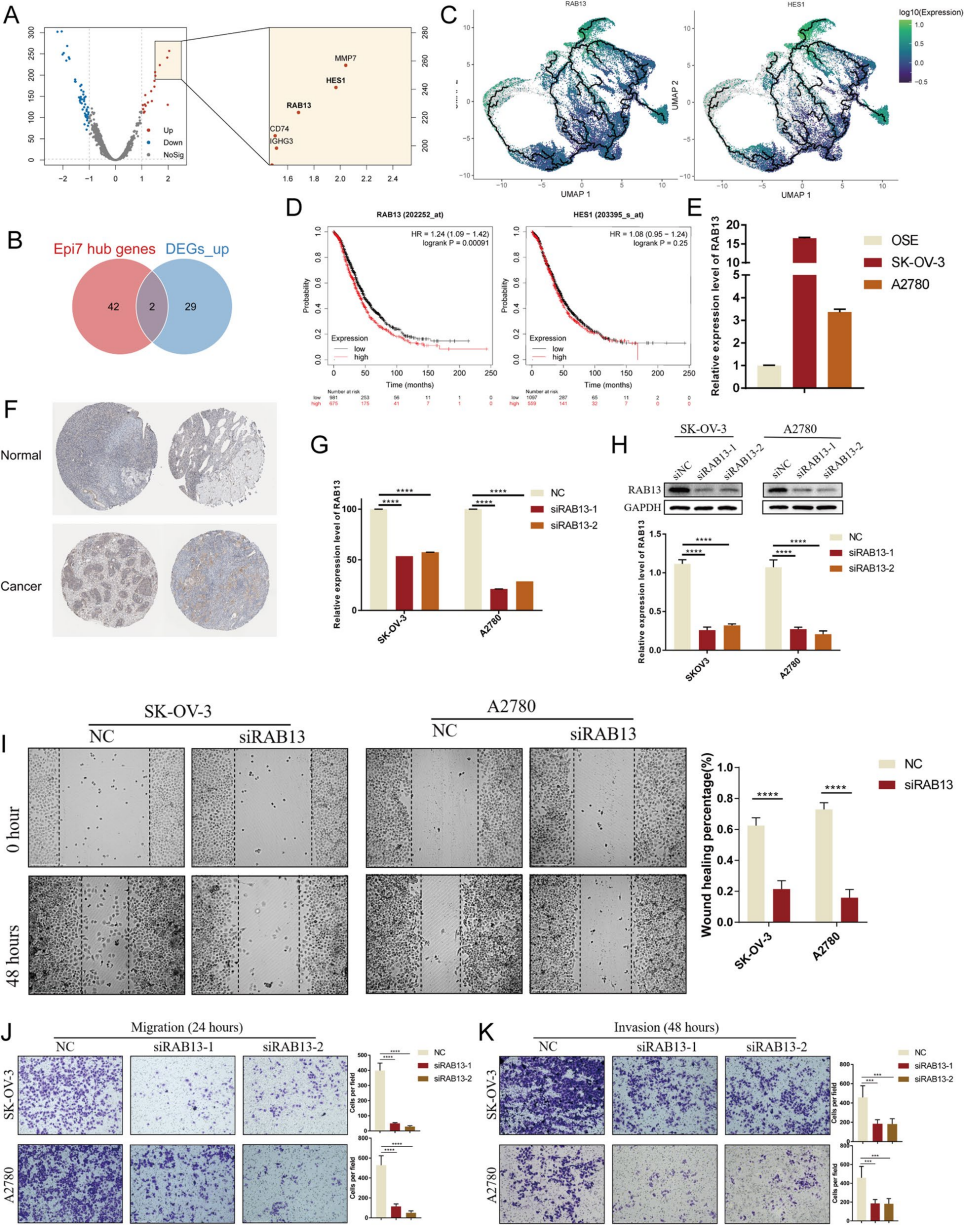
RAB13 is overexpressed in OC and promotes OC cells migration and invasion. **A** Volcano plot revealed the DEGs between primary and metastatic OC cells (left) and the five genes with the greatest difference (right). **B** Intersection of the upregulated genes in metastatic OC cells and the hub genes of Epi7. **C** RAB13 and HES1 in the UMAP plot colored by expression level with pseudotime trajectory. **D** KM curve of overall survival time and RAB13 (top) or HES1 (bottom), n = 1656. **E**, **F** The mRNA expression levels of RAB13 were higher in OC cell lines than normal ovary cell line (*p < 0.05, **p < 0.01, ***p < 0.001, ****p < 0.0001 in *t*-test. N = 3), as well as the tissue protein levels based on the Human Protein Atlas (HPA) database. **G**, **H** Western blot analysis and qRT-PCR analysis of the expression of RAB13. The expression level of protein was quantified by grey analysis. (*p < 0.05, **p < 0.01, ***p < 0.001, ****p < 0.0001 in *t*-test. N = 3). **I**, **J** The wound healing assay and Transwell migration assay showed decreased migration ability of SK-OV-3 and A2780 cell lines treated with si-RAB13. (*p < 0.05, **p < 0.01, ***p < 0.001, ****p < 0.0001 in *t*-test. N = 3). **K** The Transwell invasion assay showed decreased invasion ability of SK-OV-3 and A2780 cell lines treated with si-RAB13. (*p < 0.05, **p < 0.01, ***p < 0.001, ****p < 0.0001 in *t*-test. N = 3)

By differential expression analysis (DEA) between primary and metastatic OC cells, we identified 31 upregulated genes in metastatic cancer cells (Fig. 5A, Additional file 7: Table S3). Taking the intersection with the hub genes of Epi7, we obtained 2 genes, RAB13 and HES1 (Fig. 5B, C). KM curves plotted by Kaplan–Meier Plotter [27], demonstrated a significantly negative correlation between RAB13 and overall survival time (logrank p = 0.00091), while HES1 was not significant (Fig. 5D). We further explored the mRNA expression of RAB13 in human OC cell lines SK-OV-3, A2780 and normal ovarian cell line OSE. The RT-qPCR results showed that RAB13 was significantly upregulated in SK-OV-3 and A2780 compared with OSE (Fig. 5E). Meanwhile, we collected the immunohistochemical pictures of RAB13 in OC from HPA database [28], which showed the same trend with the mRNA levels (Fig. 5F). To verify the function of RAB13 in ovarian cancer, we detected the cellular phenotypic changes in OC cell lines that knocked down RAB13. In both SK-OV-3 and A2780 cell lines, RAB13 was efficiently knocked down by two siRNAs (Fig. 5G, H). Subsequently, we found that the knockdown of RAB13 reduced the ability of OC cells to migrate and invade by wound-healing assay and Transwell assays. (Fig. 5I–K). These results demonstrated that RAB13 plays an important role in the invasion and migration of OC cells.

### Functions of RAB13 and potential drugs prediction

**Fig. 6:**
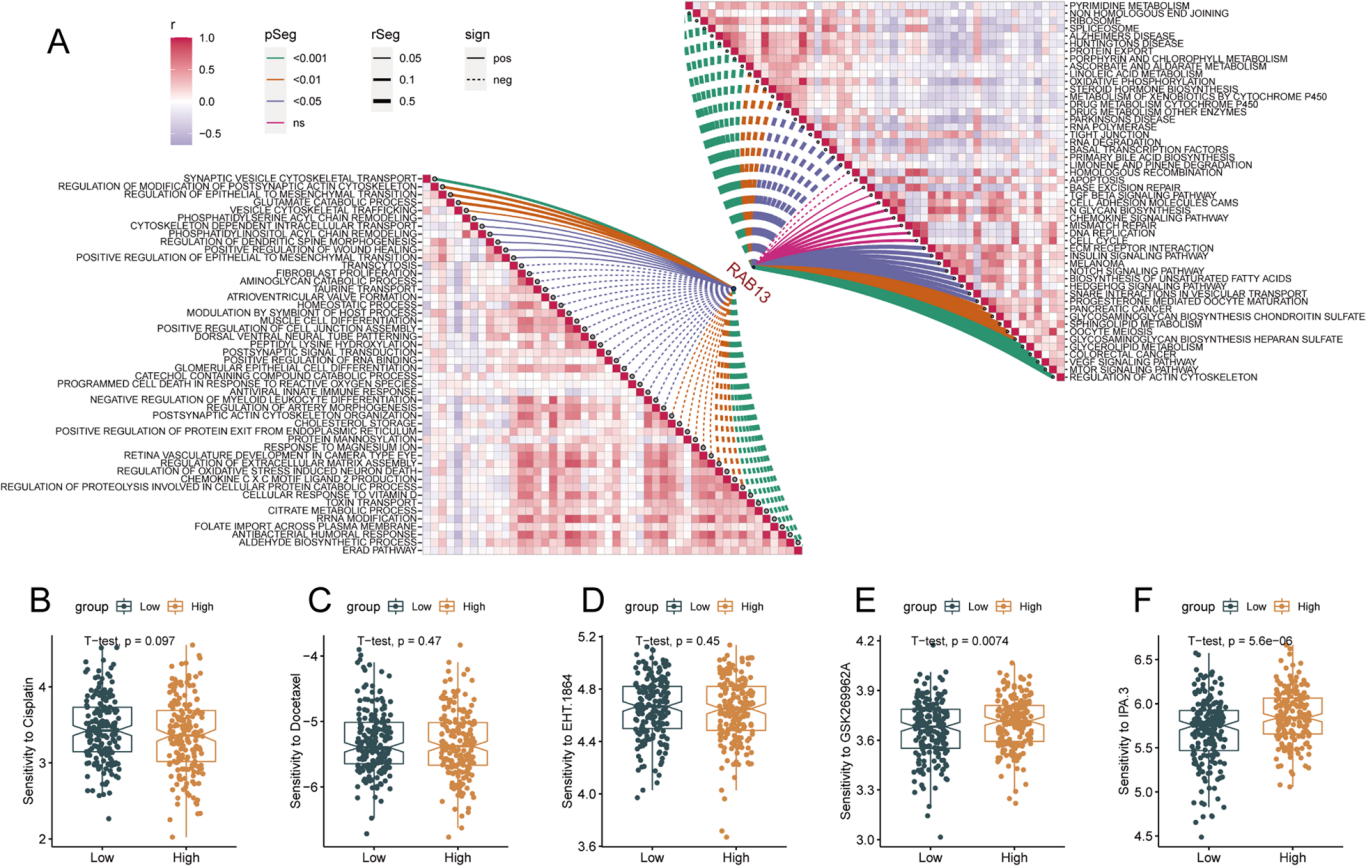
Functions and Drug Response Prediction of RAB13 based on TCGA cohort. **A** Correlations between RAB13 and the enrichment scores of GOBP (left bottom) as well as KEGG (right top) pathways. **B**–**F** Boxplots displaying the sensitivity of the conventional chemotherapy drugs (**B**, **C**) or potential cytoskeleton inhibitors (**D**–**F**) among OC patients

We then analyzed the correlations between RAB13 and the enrichment scores of TCGA patients based on GOBP and KEGG pathways (Fig. 6A). RAB13 negatively correlated with apoptosis pathways such as ‘programmed cell death in response to reactive oxygen species’. Furthermore, RAB13 positively correlated to cytoskeleton pathways and tumorigenic pathways such as ‘regulation of actin cytoskeleton’, ‘mTOR signaling pathway’, ‘VEGF signaling pathway’, ‘synaptic vesicle cytoskeletal transport’ and ‘positive regulation of wound healing’. In summary, RAB13 might promote migration and invasion through cytoskeleton pathways. Then, we explored the correlation between RAB13 and the sensitivity to conventional chemotherapy, including cisplatin and docetaxel (Fig. 6B, C), and the results were not significant. Additionally, we investigated the potential cytoskeleton inhibitors from the Genomics of Drug Sensitivity in Cancer (GDSC) based on the gene module of RAB13 (Fig. 6D–F), and found two drugs (IPA.3 and GSK269962A) with better response sensitivities for the high-expressed groups, which further revealed that our findings could help develop new therapeutic strategies for OC patients.

## Discussion

Intercellular and inter-patient heterogeneity of ovarian cancer is particularly strong due to different origins and the influence of complex biological factors [29]. Meanwhile, the heterogeneity has posed a major obstacle to the study of universal treatments for ovarian cancer and is one of the most important reasons for treatment failure, metastasis and recurrence of OC [30]. Single-cell sequence technologies provide us a new perspective to study tumor cell clusters and intracellular interactions.

In this study, we explored the heterogeneity within ovarian cancer, especially between primary and metastatic cancer cells, and we found that there were heterogeneous subclusters within cancer cells. Such as C7, whose genetic signature was associated with a better prognosis, although they originate mainly from metastatic tumors. Since its functional analysis showed that it was associated with immune response related pathways, we suggested that it was in a low immune escape state. In addition, C5 essentially originated from metastatic tumors in a single patient, indicating that therapeutic measures targeting this cell subcluster would be more difficult to be widely used. Unlike C5, we found another subcluster, C4, derived from multiple patients and was significantly associated with worse prognosis. The functions of C4 mainly focused on pathways including EMT, Hedgehog signaling, angiogenesis, and cytoskeleton, which were usually considered being associated with tumor progression and metastasis [31–33]. These findings led us to focus our attention on two more specific subclusters, C4 and C7.

We also characterized a cell–cell communication atlas among malignant cells and TME cells based on receptor-ligand pairs. We found that C7 communicated with immune cells, while C4 hardly communicated with immune cells, indicating it might be an immune escaped cell cluster. Meanwhile, the interactions of C4 to mesenchymal cells were much more than those in other malignant cells. Pathways including VEGF, Laminin and Collagen contributed significantly to the communication. The VEGF pathway has closely associated with angiogenesis and tumor invasion [34]. The laminin and collagen pathways correlated to ECM remodeling, as the tumor cells degrade ECM to make a path to travel to distant places [26, 35]. Therefore, it is suggested that the malignant cells in C4 are the driving population in tumor invasion and migration.

We further revealed the gene modules in C4 and identified its hub genes named RAB13 (RAB13, Member RAS Oncogene Family) and HES1 (Hes Family BHLH Transcription Factor 1). As a downstream target gene of NOTCH signaling pathway, the role of HES1 in cancer has been extensively studied. HES1 has been shown to promote cell invasion via STAT3-MMP14 pathway and is associated with poor prognosis in colorectal cancer [36]. However, in this study, we found that HES1 was not associated with the prognosis of OC. Furthermore, the pathway analysis results indicated that the NOTCH signaling pathway was not the most critical pathway in the C4 subcluster. Therefore, we shifted our focus to another gene, RAB13. It is a member of the Rab family of small G proteins and plays an important role in cell–cell junctions, which was associated with tumor progression and metastasis in breast and cervical cancer [37, 38].

**Fig. 7:**
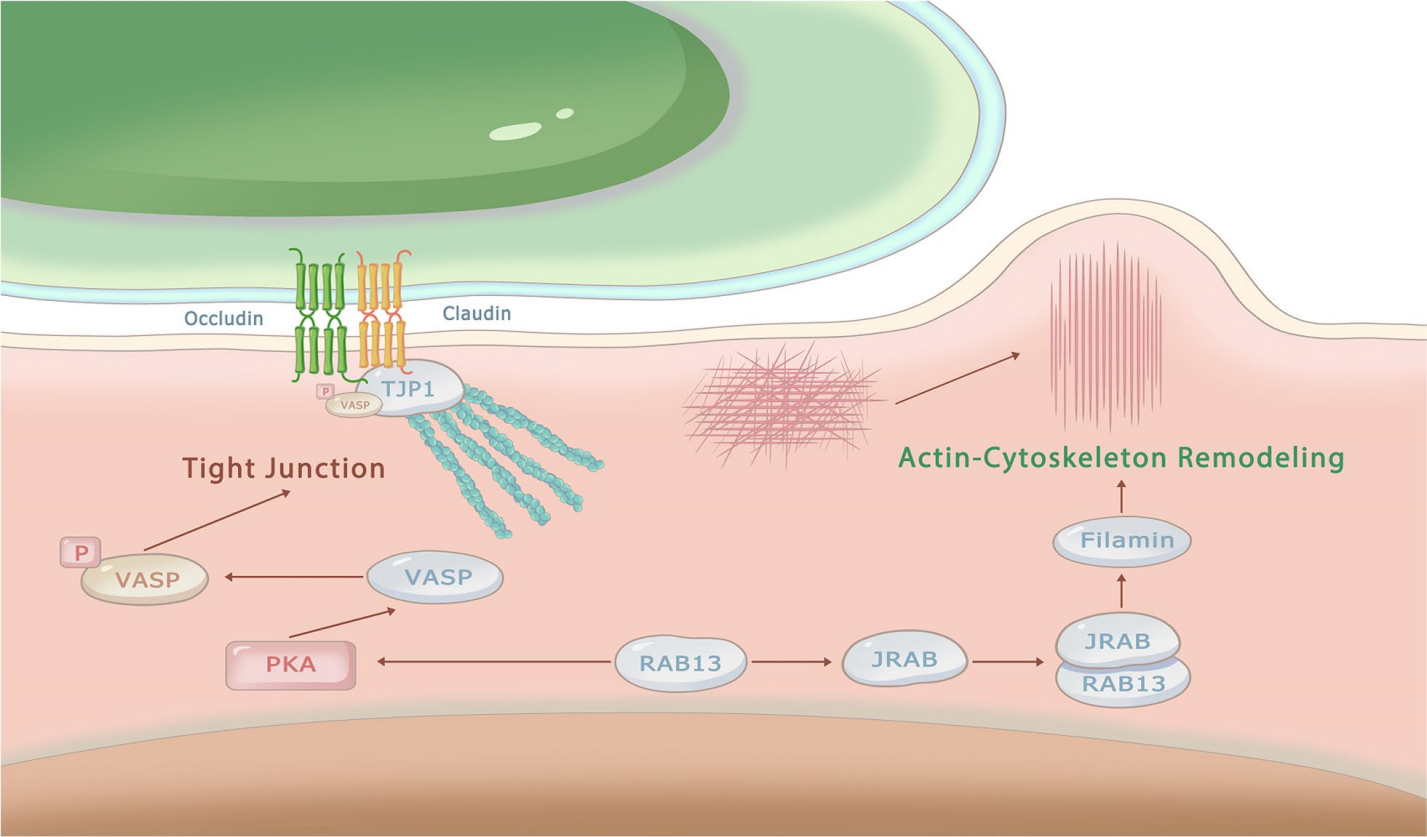
RAB13 promotes cell migration via regulating the tight junction and actin cytoskeleton. RAB13 inhibits the phosphorylation of VASP and suppress the binding of TJP1 with claudin and occludin by suppressing the ability of PKA, leading to decreased cell adhesion; Meanwhile, RAB13 forms a complex with JRAB and inhibits the remodeling of cytoskeleton by affecting filamin, thereby promoting the migration ability of cancer cells

Our study found that RAB13 plays a significant role in regulating pathways related to cytoskeleton remodeling and tight junctions (Fig. 6A), which are known to affect the movement and migration of cells [39–41]. Katja et al. found that RAB13 inhibits protein kinase 1 to suppress the phosphorylation of vasodilator-stimulated phosphoprotein (VASP), and the phosphorylated VASP is necessary for the transmembrane proteins claudin and occludin to attach tight junctions to the actin cytoskeleton via binding with tight junction protein 1 [39, 42]. Moreover, Ayoku et al. demonstrated that RAB13 could form a complex with its downstream effector RAB13 binding protein (JRAB), which recruits the actin cytoskeleton-binding protein filamin to the site [40, 41]. Filamin promotes the formation of membrane protrusions and leads to directed cell migration [43]. Based on these studies, we suggest that RAB13 may enhance cell migration ability through two closely related mechanisms as shown in Fig. 7. However, the effects of RAB13 in OC have been hardly reported yet. In our study, we validated the effect of RAB13 for the first time in OC, and demonstrated its role in promoting OC cell migration and invasion. Consistently with previous hypothesis, molecular functions of RAB13 in OC were indicated to correlate with cell movement and migration, since the enrichment results showed pathways positively related to cytoskeleton and negatively related to tight junction. This provides a basis for further exploration of the potential value of RAB13 in the treatment of ovarian cancer.

Furthermore, RAS family, has a mutation rate of 15% in OC, and these mutations can lead to metabolic reprogramming, which is one of the most important hallmarks of cancer cells [44]. Metabolic reprogramming occurs through multiple metabolic changes such as in glucose, fatty acid and glutamine, which can affect the interactions between cancer cells and TME, leading to malignant biological process including cell migration, angiogenesis and immune escape [45]. As findings in this study (Fig. 4H), metabolic pathways play an important role in C4, which is the driving cell cluster of OC metastasis. The functions of RAB13 are also enriched in metabolic pathways such as Glycerolipid Metabolism, Sphingolipid Metabolism (Fig. 6A). However, as a member of RAS family, the character of RAB13 in cancer metabolism has been rarely discussed, especially in OC. This would be an interesting research direction, such as studying the metabolomics of OC cells or patients grouped by RAB13 expression level and detecting the differential expressed metabolites and genes by mass spectrometry and next-generation sequence.

In addition to conventional treatments including platinum and paclitaxel chemotherapy, new drugs such as PARP inhibitors and VEGF inhibitors have been introduced to clinical use and have significantly improved the prognosis of many OC patients [46, 47]. However, despite improvements for the therapeutic methods, the prognosis of most OC patients still remains poor [46]. Here, we aimed to find an approach that will benefit most patients. Since C4 is a cell cluster with prevalence among OC patients, we suggested that its marker RAB13 might be a new therapeutic target. We further predicted the sensitivity of several compounds targeting cytoskeleton and found two of them were significant including IPA.3 and GSK269962A, which may provide new options for the treatment of OC.

## Conclusions

This study provided a new perspective on understanding the progression and metastasis of OC. Furthermore, we revealed a specific cell cluster associated with a high propensity to metastasize and its marker, RAB13 has great potential to become a new therapeutic target for OC.

## Supplementary Information

**Additional file 1: Figure S1.** UMAP of integrated data identified 18 cell clusters.

**Additional file 2: Figure S2.** Chromosomal landscape of inferred CNVs among malignant OC cell clusters.

**Additional file 3: Figure S3.** Kaplan–Meier analysis for patients from TCGA cohort with high and low GSVA score based on the top 20 markers of 7 cell subclusters.

**Additional file 4: Figure S4.** The rank of pathways contribution to cell–cell communication.

**Additional file 5: Table S1.** Clinical data of 14 samples used in the single-cell sequence analyses.

**Additional file 6: Table S2.** Genes were arranged in descending order of the morans_I calculated by pseudotime analysis.

**Additional file 7: Table S3.** Differentially expressed genes between primary and metastatic cancer cells with their log_2_FoldChange and p-value.

## Data Availability

The code used in our study can be obtained from the corresponding author. The data sets used in the present research were summarized in the Additional file 5: Table S1 and the single-cell raw data have been deposited in the SRA data sets (PRJNA756768).
