# Supplementary figures and images for: Single-cell transcriptomics in ovarian cancer identify a metastasis-associated cell cluster overexpressed RAB13
Source: J Transl Med. 2023 Apr 12;21:254. doi: 10.1186/s12967-023-04094-7 (PMC10091580; doi:10.1186/s12967-023-04094-7)

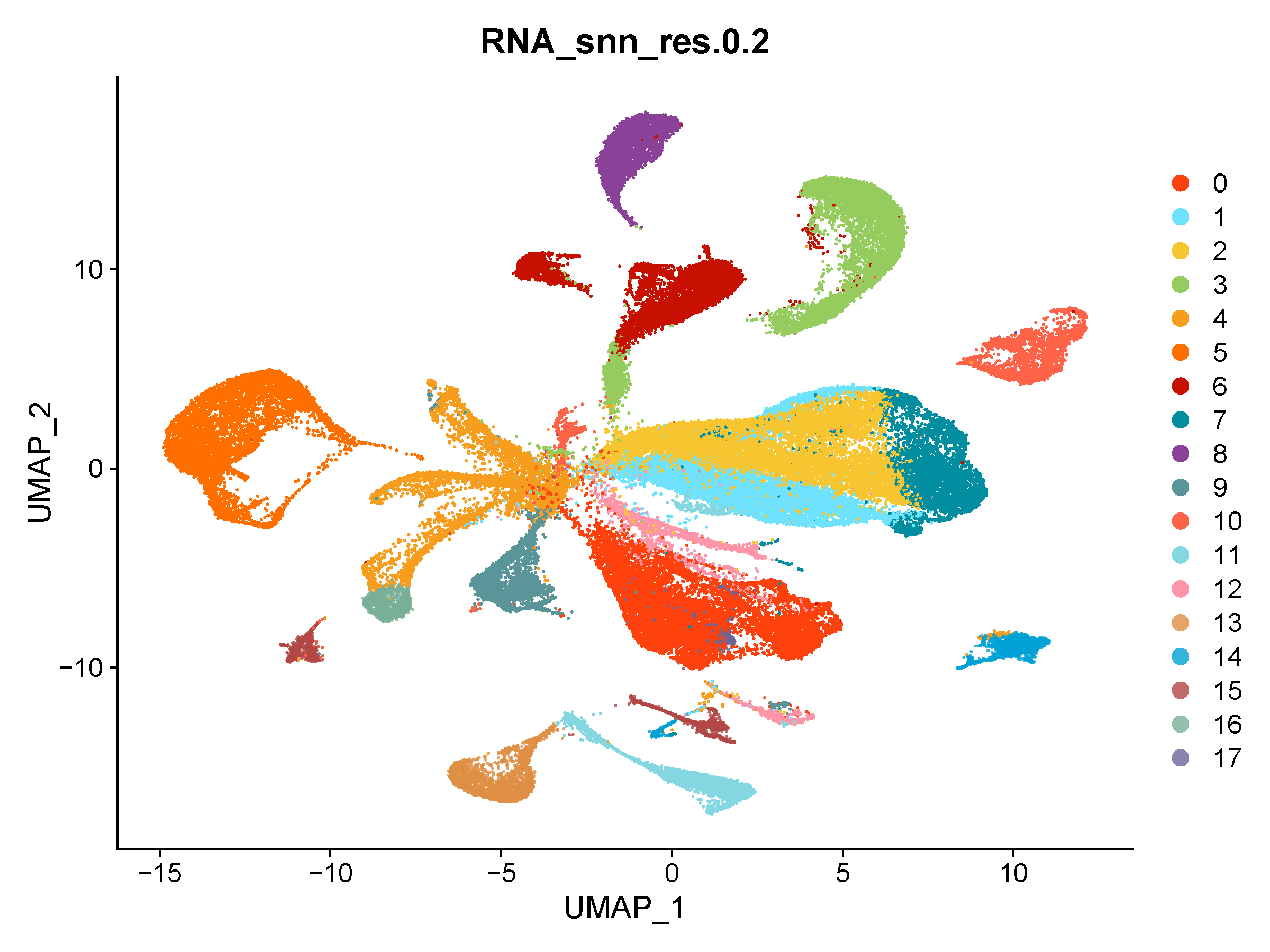

Supplement: Supplementary file 1 — Additional file 1: Figure S1. UMAP of integrated data identified 18 cell clusters. [file 12967_2023_4094_MOESM1_ESM.jpg]

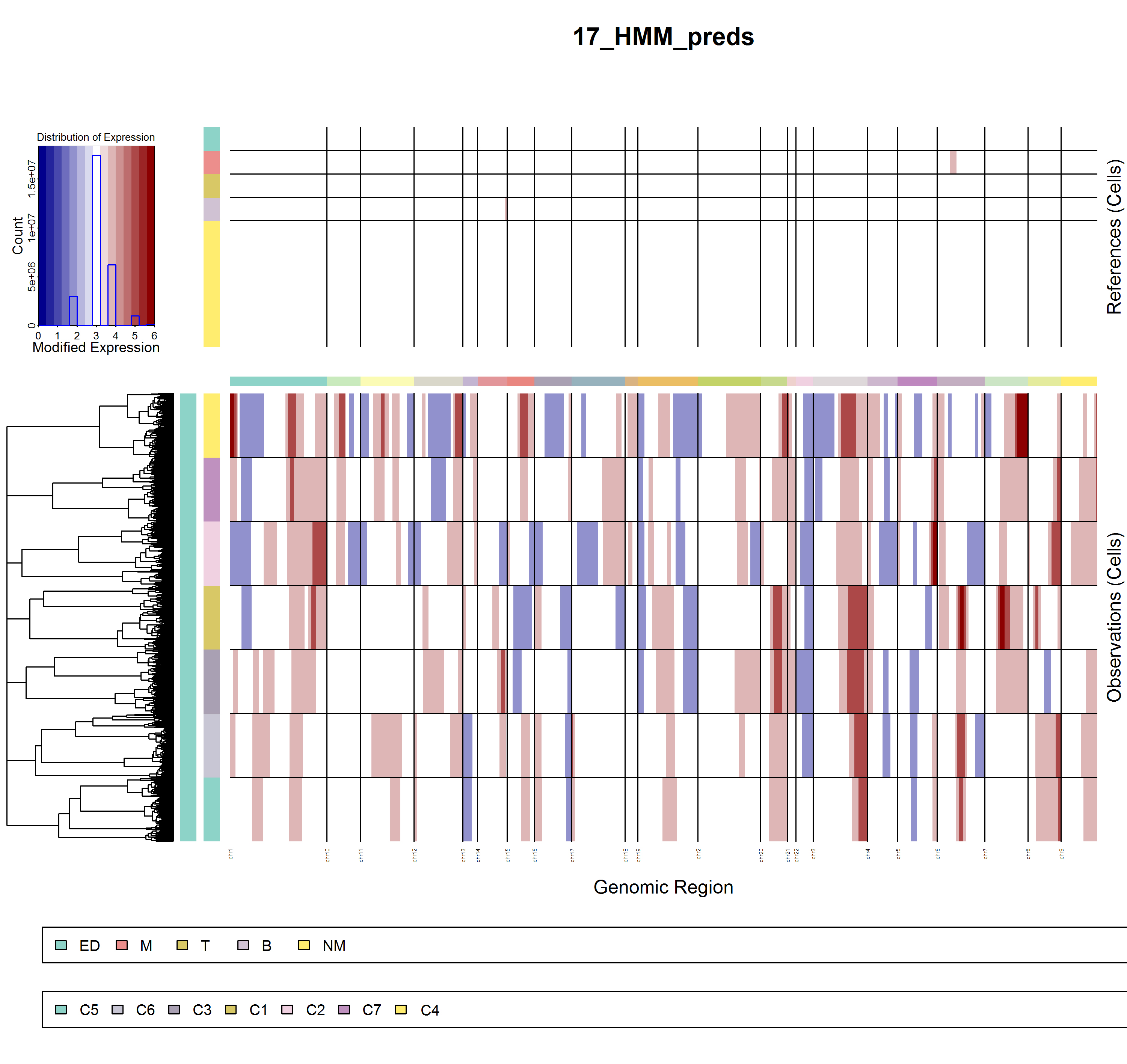

Supplement: Supplementary file 2 — Additional file 2: Figure S2. Chromosomal landscape of inferred CNVs among malignant OC cell clusters. [file 12967_2023_4094_MOESM2_ESM.jpg]

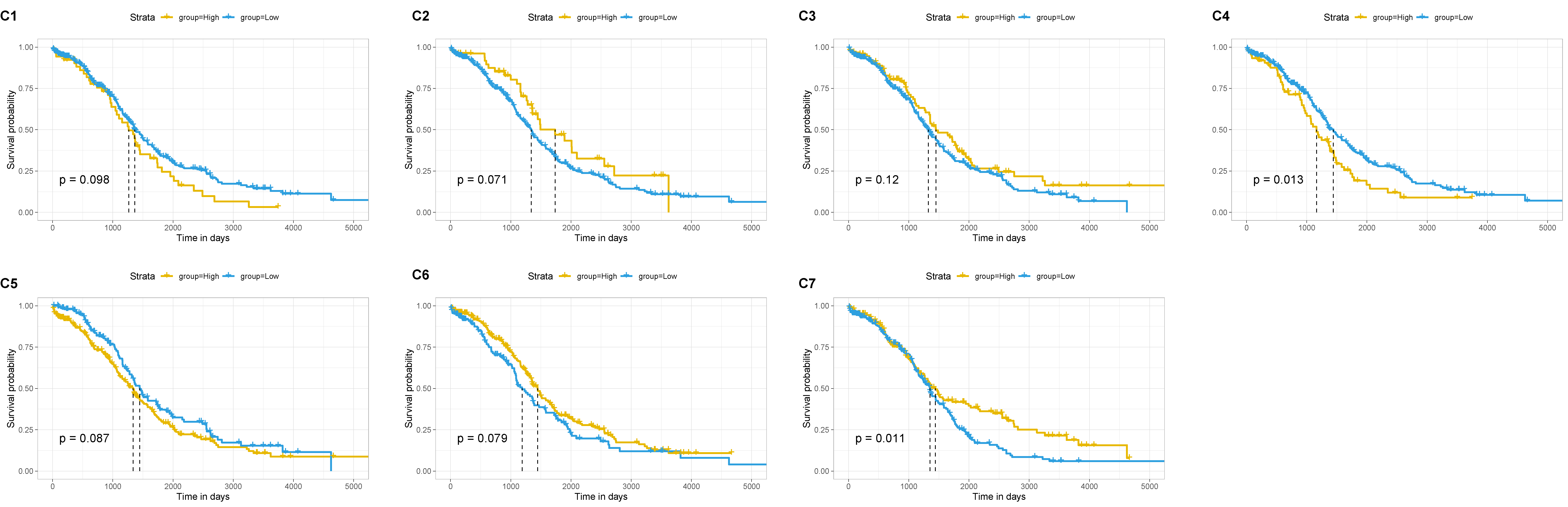

Supplement: Supplementary file 3 — Additional file 3: Figure S3. Kaplan–Meier analysis for patients from TCGA cohort with high and low GSVA score based on the top 20 markers of 7 cell subclusters. [file 12967_2023_4094_MOESM3_ESM.jpg]

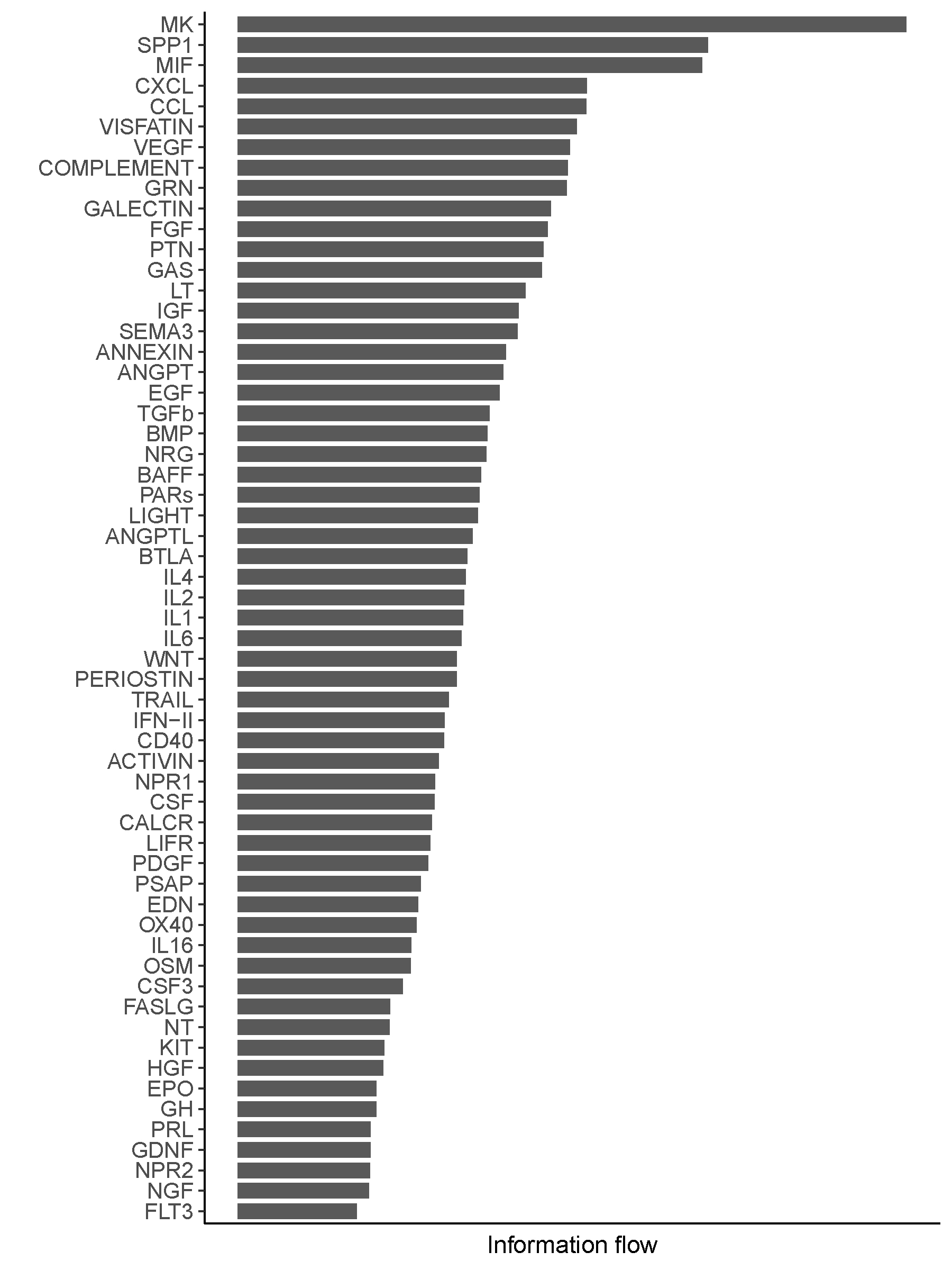

Supplement: Supplementary file 4 — Additional file 4: Figure S4. The rank of pathways contribution to cell–cell communication. [file 12967_2023_4094_MOESM4_ESM.jpg]
