# Supplementary material for: Single-cell transcriptomics in ovarian cancer identify a metastasis-associated cell cluster overexpressed RAB13
Source: J Transl Med. 2023 Apr 12;21:254. doi: 10.1186/s12967-023-04094-7 (PMC10091580; doi:10.1186/s12967-023-04094-7)
Supplement: Supplementary file 5 — Additional file 5: Table S1. Clinical data of 14 samples used in the single-cell sequence analyses. [file 12967_2023_4094_MOESM5_ESM.docx]

**Table S1.**

**Clinical characteristics of OC patients and control sample**

| Patient ID | Pathologic diagnoses | Tissue source | FIGO Stage | Access number |
| --- | --- | --- | --- | --- |
| NM1 | Normal ovary control | Ovary | N/A | PRJNA756768 |
| PT1 | HGSOC | Ovary | IIIc | PRJNA756768 |
| PT2 | HGSOC | Ovary | N/A | GSE181955 |
| PT3 | HGSOC | Ovary | N/A | GSE181955 |
| PT4 | HGSOC | Ovary | N/A | GSE158937 |
| PT5 | HGSOC | Ovary | N/A | GSE158937 |
| PT6 | HGSOC | Omentum | N/A | GSE158937 |
| MT1 | HGSOC | Omentum | N/A | GSE181955 |
| MT2 | HGSOC | Omentum | N/A | GSE181955 |
| MT3 | HGSOC | Omentum | IV | GSE154600 |
| MT4 | HGSOC | Omentum | IIIc | GSE154600 |
| MT5 | HGSOC | Omentum | IVb | GSE154600 |
| MT6 | HGSOC | Omentum | IV | GSE154600 |
| MT7 | HGSOC | Omentum | IVa | GSE154600 |

HGSOC: High grade serous ovarian cancer
